# Supplementary figures and images for: Mediator MED23 regulates inflammatory responses and liver fibrosis
Source: PLoS Biol. 2019 Dec 5;17(12):e3000563. doi: 10.1371/journal.pbio.3000563 (PMC6917294; doi:10.1371/journal.pbio.3000563)

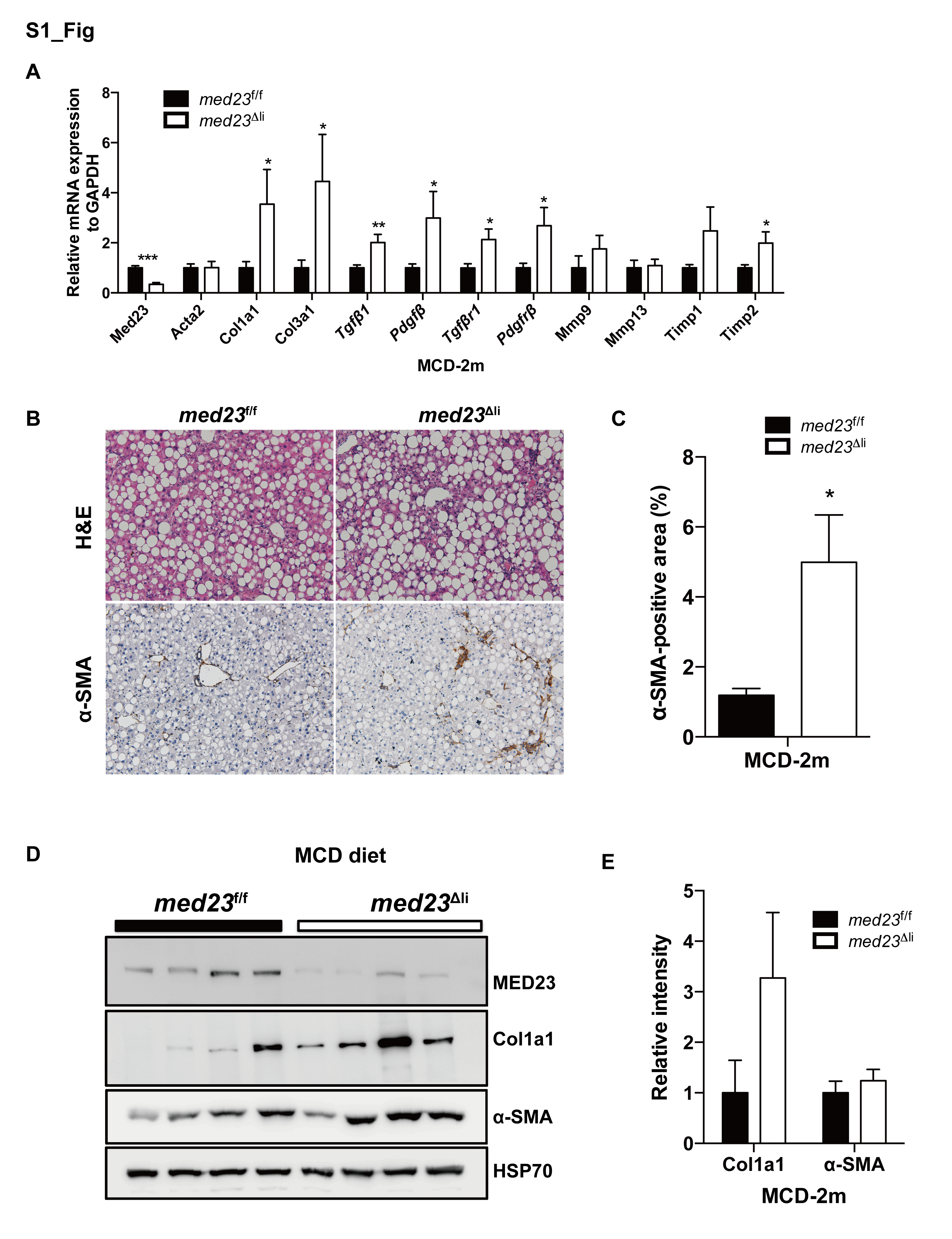

Supplement: S1 Fig — (A) qRT-PCR analysis of fibrosis-related factors in whole-liver extracts of med23f/f and med23Δli mice fed with a MCD diet (med23f/f, n = 9; med23Δli, n = 6). (B) Liver sections from med23f/f and med23Δli mice fed with a MCD diet were stained with HE and α-SMA, and representative views are shown. (C) Quantification of the α-SMA-positive area in livers of med23f/f and med23Δli mice (med23f/f, n = 9; med23Δli, n = 6). (D) The total protein was extracted from whole livers of med23f/f and med23Δli mice fed with a MCD diet and analyzed by western blotting using the indicated antibodies. HSP70 was used as a loading control. (E) Quantification of the α-SMA and Col1a1 levels in livers of med23f/f and med23Δli mice (by the gray degree value in D) and normalized to the HSP70. Error bars denote SEM from three independent experiments (med23f/f, n = 4; med23Δli, n = 4). Data are presented as means ± SEM. *P < 0.05, **P < 0.01, ***P < 0.001. For underlying data, see S1 Data file. α-SMA, alpha-smooth muscle actin; Col, collagen; HE, hematoxylin–eosin; HSP70, hot shock protein 70; MCD, methionine and choline-deficient; med23, Mediator complex subunit 23; med23Δli, liver-specific knockout of Med23; med23f/f, med23-floxed; qRT-PCR, quantitative real-time PCR. (TIF) [file pbio.3000563.s001.tif]

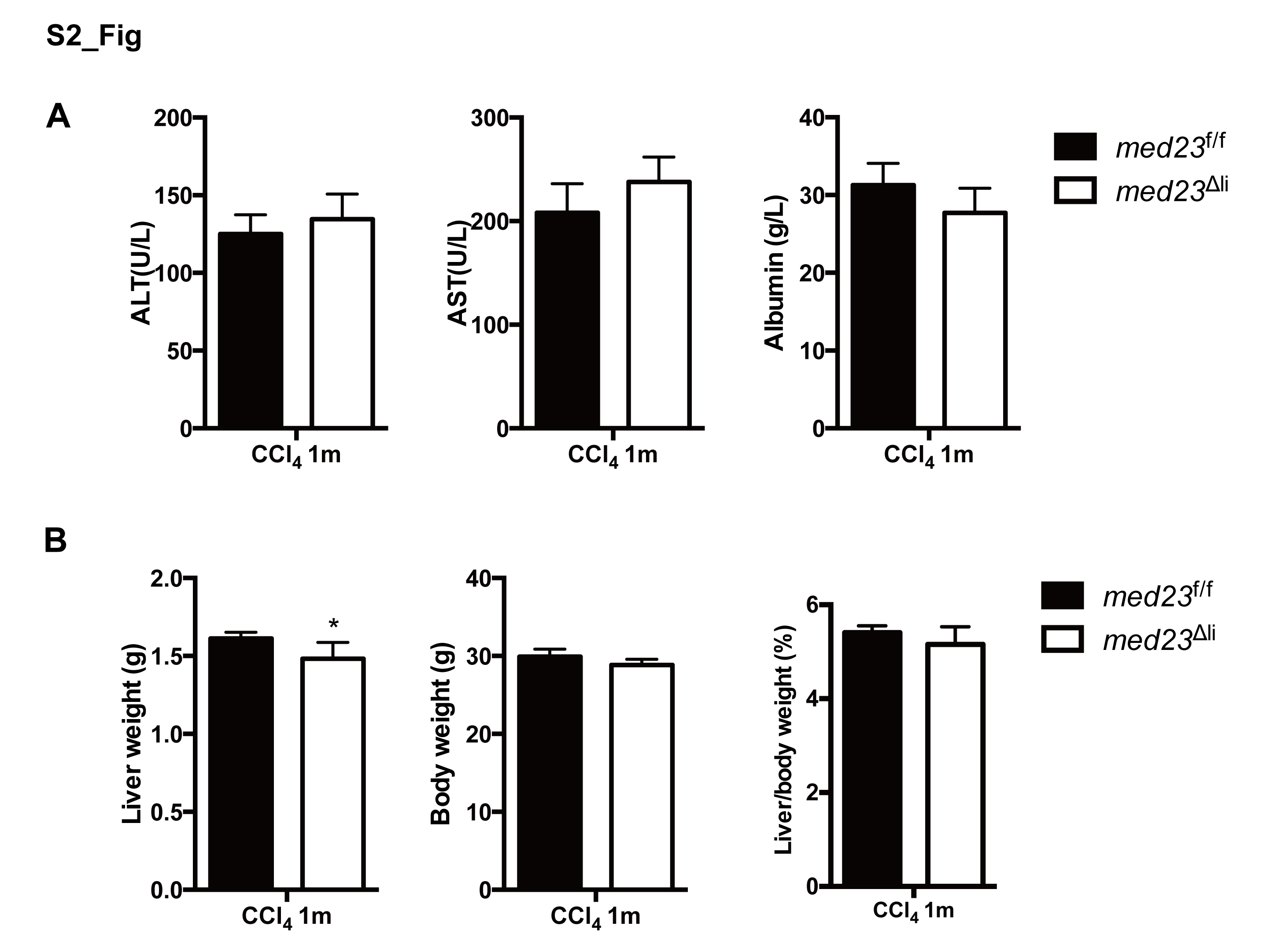

Supplement: S2 Fig — (A) Serum ALT, AST, and albumin were measured in med23f/f and med23Δli mice (n = 6–7 per group). (B) Analysis of liver weight, body weight, and liver/body weight in med23f/f and med23Δli mice (n = 7 per group). Data are presented as means ± SEM. *P < 0.05, **P < 0.01. For underlying data, see S1 Data file. ALT, alanine aminotransferase; AST, aspartate aminotransferase; CCl4, carbon tetrachloride; med23, Mediator complex subunit 23; med23Δli, liver-specific knockout of Med23; med23f/f, med23-floxed. (TIF) [file pbio.3000563.s002.tif]

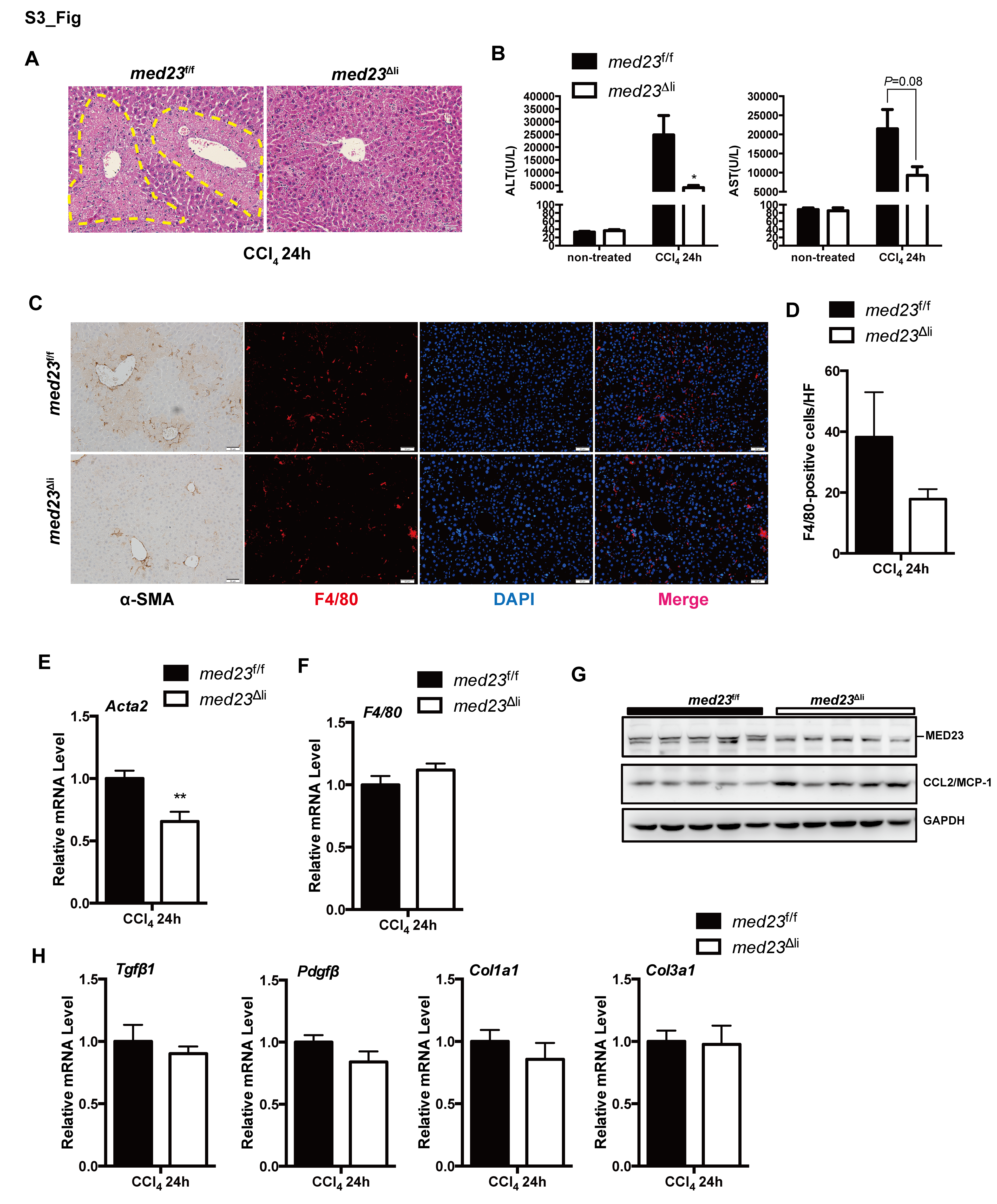

Supplement: S3 Fig — (A) Representative views of HE staining in the liver sections of med23f/f and med23Δli mice after acute CCl4 administration for 24 hours. (B) Serum ALT and AST were measured in med23f/f and med23Δli mice after acute CCl4 administration for 24 hours (nontreated: med23f/f, n = 7; med23Δli, n = 7; CCl4 24h: med23f/f, n = 5; med23Δli, n = 4). (C) Representative images of α-SMA and F4/80 staining in the liver sections of med23f/f and med23Δli mice after acute CCl4 administration for 24 hours. The liver paraffin sections were stained with α-SMA antibody, and liver frozen sections were stained with F4/80 antibody (red). DAPI (blue) was used for nuclear counterstaining. (D) Quantification of F4/80-positive cells from the liver sections of med23f/f and med23Δli mice (med23f/f, n = 6; med23Δli, n = 4). (E-F) qRT-PCR analysis of Acta2 (E) and F4/80 (F) expression in whole-liver extracts of med23f/f and med23Δli mice. The mRNA expression was normalized to Gapdh (med23f/f, n = 6; med23Δli, n = 5). (G) Western blotting analysis of total protein extracted from whole livers of med23f/f and med23Δli mice using the indicated antibodies. GAPDH was used as a loading control. (H) qRT-PCR analysis of fibrosis-associated genes expression in whole-liver extracts of med23f/f and med23Δli mice (med23f/f, n = 6; med23Δli, n = 5). Data are presented as means ± SEM. **P < 0.01. For underlying data, see S1 Data file. α-SMA, alpha-smooth muscle actin; ALT, alanine aminotransferase; AST, aspartate aminotransferase; CCl4, carbon tetrachloride; GAPDH, glyceraldehyde 3-phosphate dehydrogenase; HE, hematoxylin–eosin; med23, Mediator complex subunit 23; med23Δli, liver-specific knockout of Med23; med23f/f, med23-floxed; qRT-PCR, quantitative real-time PCR. (TIF) [file pbio.3000563.s003.tif]

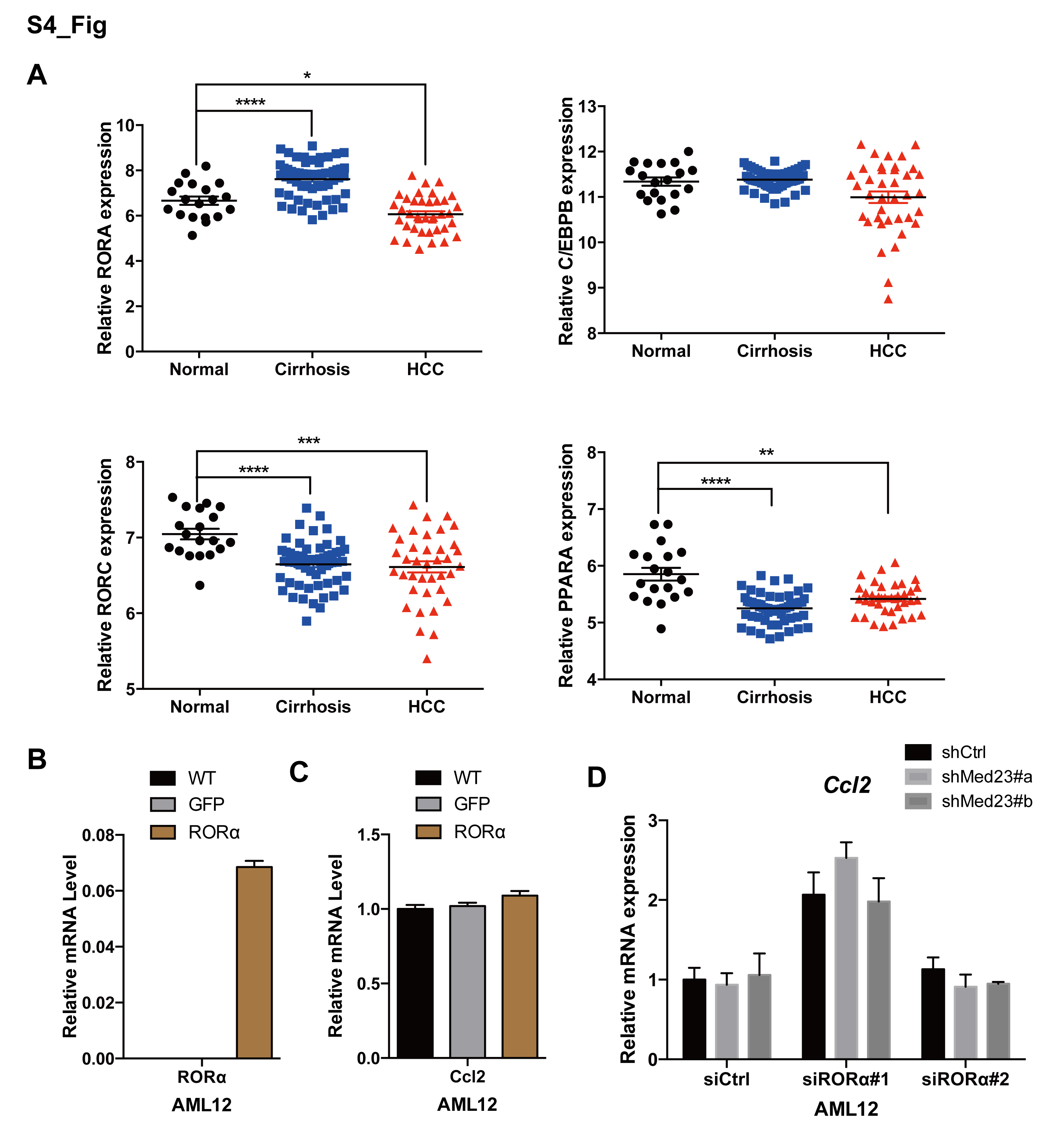

Supplement: S4 Fig — (A) The published database (GSE14323) [48] was utilized to evaluate the mRNA expression of predicted upstream regulators (normal, n = 19; cirrhosis, n = 58; HCC, n = 38). (B-C) qRT-PCR analysis of RORα (B) and Ccl2 (C) expression in AML12 cells after RORα overexpression. The mRNA expression was normalized to Gapdh (n = 4 per group). (D) qRT-PCR analysis of Ccl2 expression in shCtrl and shMed23 AML12 cells after RORα knockdown. The expression was normalized to Gapdh (n = 3 per group). *P < 0.05, **P < 0.01, ***P < 0.001, ****P < 0.0001. For underlying data, see S1 Data file. AML12, alpha mouse liver 12; Ccl, C-C motif chemokine ligand; Gapdh, glyceraldehyde 3-phosphate dehydrogenase; HCC, hepatocellular carcinoma; MED23, Mediator complex subunit 23; qRT-PCR, quantitative real-time PCR; RORα, RAR-related orphan receptor alpha; shCtrl, negative control vector containing scrambled shRNA; shMed23, shRNA against Med23. (TIF) [file pbio.3000563.s004.tif]

Fig 1C

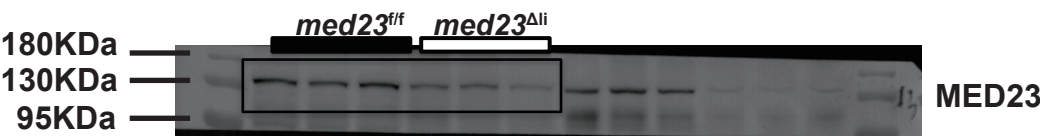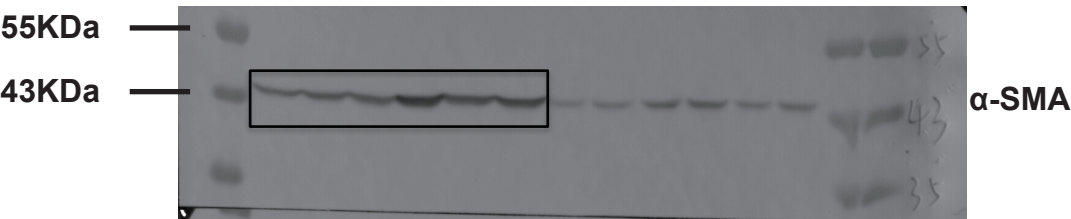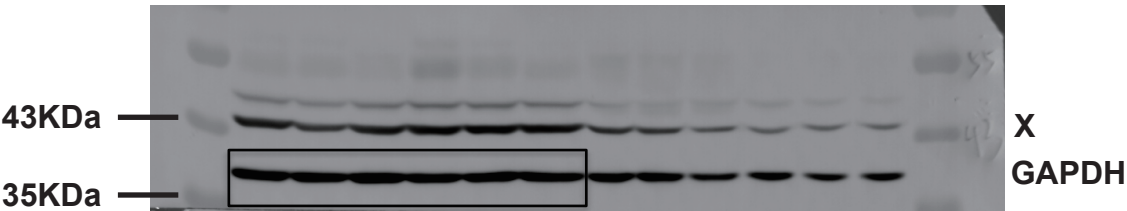

Fig 4C

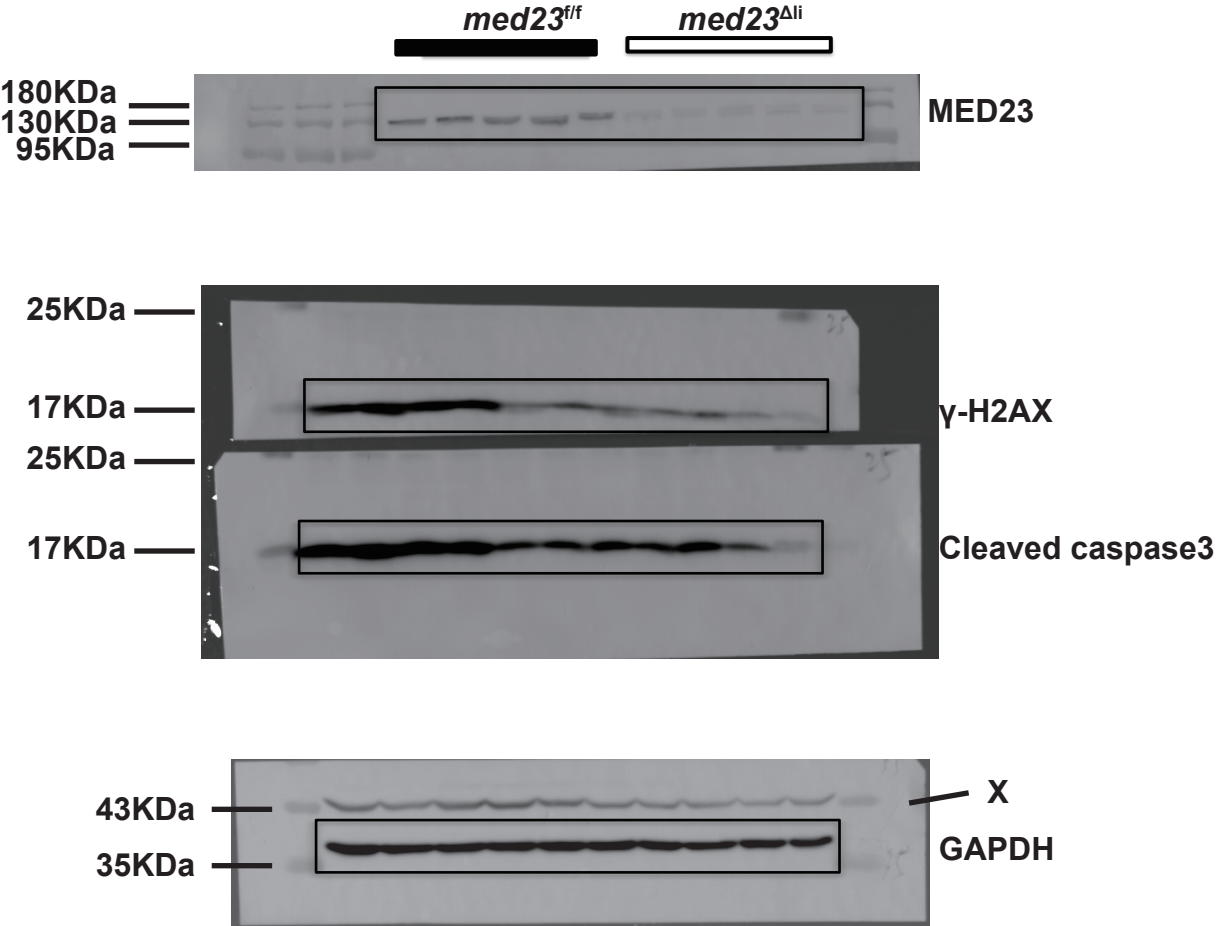

**Fig 5F**

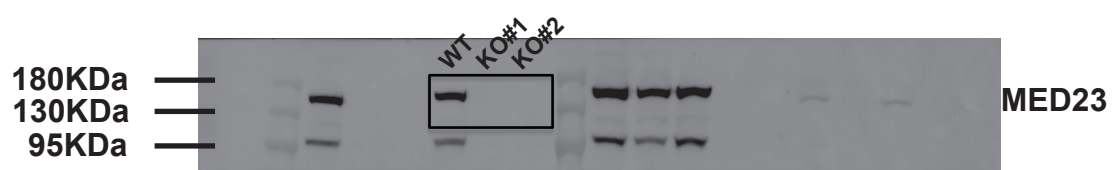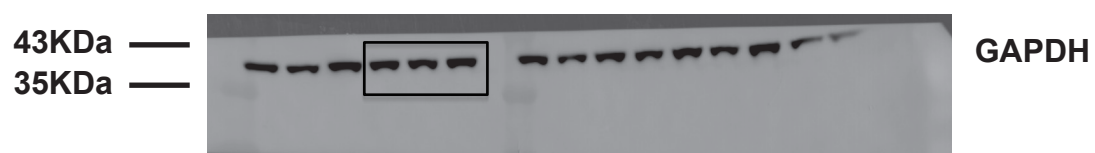

Fig 6F

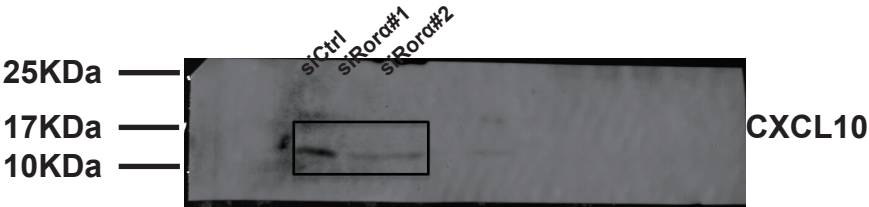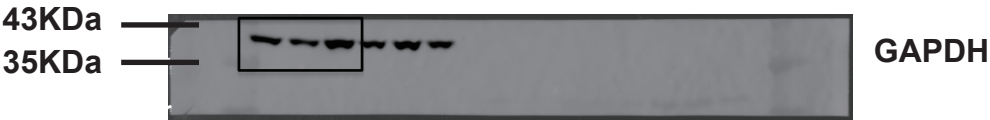

S1G Fig

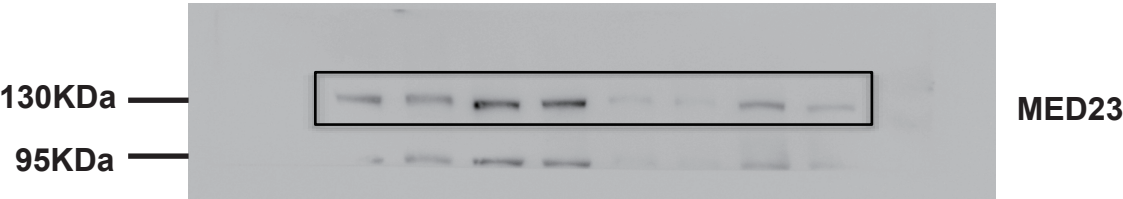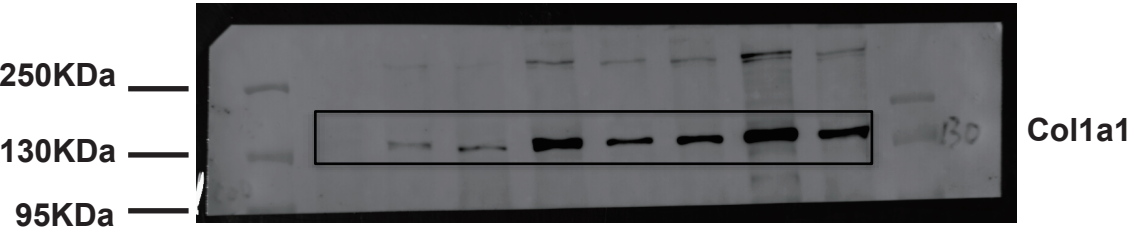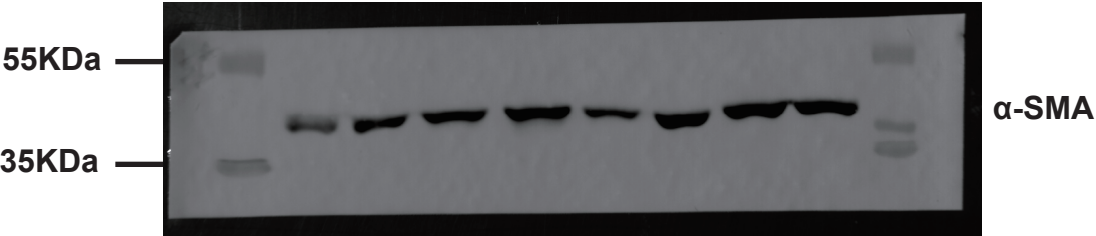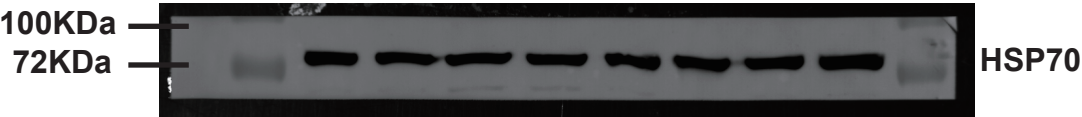

S3G Fig

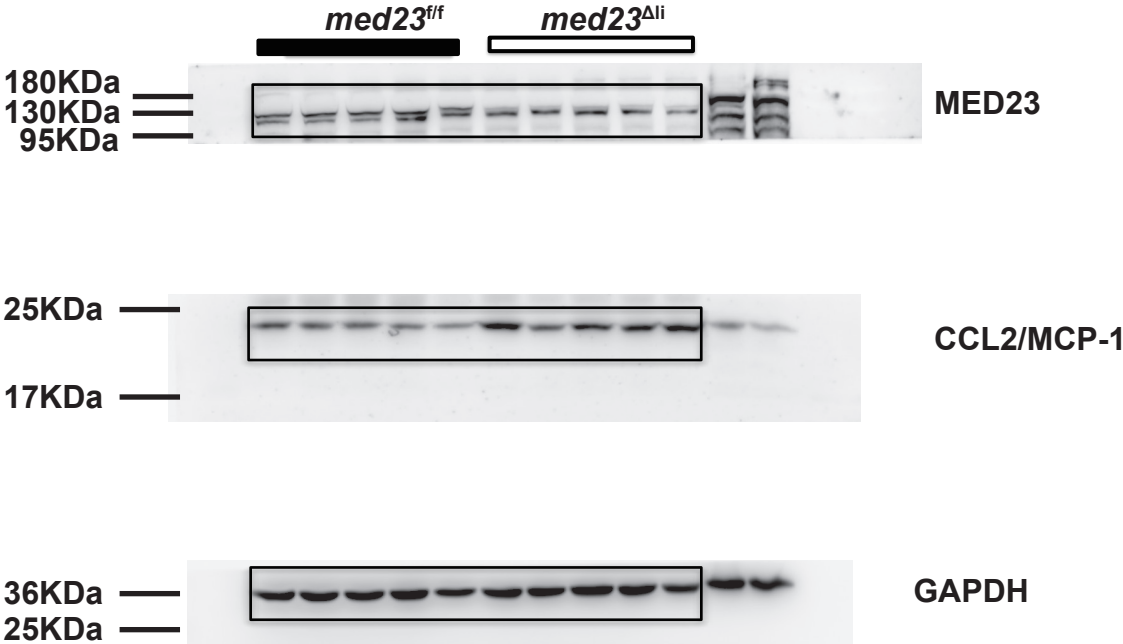

Supplement: S1 Raw Images — (PDF) [file pbio.3000563.s013.pdf]
