# Supplementary material for: Mediator MED23 regulates inflammatory responses and liver fibrosis
Source: PLoS Biol. 2019 Dec 5;17(12):e3000563. doi: 10.1371/journal.pbio.3000563 (PMC6917294; doi:10.1371/journal.pbio.3000563)
Supplement: S1 Table — shRNA, short hairpin RNA. (DOCX) [file pbio.3000563.s008.docx]

**S1 Table. The sequences of shRNA oligonucleotides:**

| **shRNA** | **sequences** |
| --- | --- |
| **shCtrl** | **GTGCGCTGCTGGTGCCAAC** |
| **shMed23#a** | **TGAAGCCCAGGTTTGTTAT** |
| **shMed23#b** | **TGGCAGTTGAGACTGGTCT** |
