# Supplementary material for: Mediator MED23 regulates inflammatory responses and liver fibrosis
Source: PLoS Biol. 2019 Dec 5;17(12):e3000563. doi: 10.1371/journal.pbio.3000563 (PMC6917294; doi:10.1371/journal.pbio.3000563)
Supplement: S2 Table — siRNA, small interfering RNA. (DOCX) [file pbio.3000563.s009.docx]

**S2 Table. The sequences of siRNA oligonucleotides:**

| siRNA | sense (5’-3’) | antisense (5’-3’) |
| --- | --- | --- |
| siCtrl | UUCUCCGAACGUGUCACGUTT | ACGUGACACGUUCGGAGAATT |
| Rora-Mus-#1 | GCAAGAUCUGUGGAGACAATT | UUGUCUCCACAGAUCUUGCTT |
| Rora-Mus-#2 | GGAAUUGUUCACUUCAGAATT | UUCUGAAGUGAACAAUUCCTT |
