# Supplementary material for: Mediator MED23 regulates inflammatory responses and liver fibrosis
Source: PLoS Biol. 2019 Dec 5;17(12):e3000563. doi: 10.1371/journal.pbio.3000563 (PMC6917294; doi:10.1371/journal.pbio.3000563)
Supplement: S3 Table — ChIP-qPCR, chromatin immunoprecipitation followed by quantitative PCR. (DOCX) [file pbio.3000563.s010.docx]

**S3 Table. Primer sequences used in the ChIP-qPCR analysis**

| Primer name | Sequence (5’-3’) |
| --- | --- |
| *Ccl5*-pro-1-F | GCAGTTAGAGGCAGAGTCATAC |
| *Ccl5*-pro-1-R | CCAGGGTAGCAGAGGAAGTG |
| *Ccl5*-pro-2-F | CAAACAGACAGACAGACAGACAA |
| *Ccl5*-pro-2-R | TGTTGTAAGGAATTTGCCAGGT |
| *Ccl5*-pro-3-F  *Ccl5*-pro-3-R  *Ccl5*-pro-4-F  *Ccl5*-pro-4-R  *Ccl5*-pro-5-F  *Ccl5*-pro-5-R | CTGCTACTCCATCCCAGAGC  ACCACATCCACCTTGTCCAT  TACAGAGCACAAGGCACAGG  AGAGGTAGGCAAAGCAGCAG  TTTCTTTGAGGACACCTGCTC  GTTGTTGCTCTTGGCATTCT |
| *Cxcl10*-pro-1-F | TCTTTCCCAAGCAGCAACTC |
| *Cxcl10*-pro-1-R | ACCCAAGGCAGCAGATACC |
| *Cxcl10*-pro-2-F | AATGCCCTCGGTTTACAGG |
| *Cxcl10*-pro-2-R  *Cxcl10*-pro-3-F  *Cxcl10*-pro-3-R  *Cxcl10*-pro-4-F  *Cxcl10*-pro-4-R  *Cxcl10*-pro-5-F  *Cxcl10*-pro-5-R | GTTGGCTCGGGATGTCTCT  CACACCTCACTCTTCTGTTTCG  CTCTCTCTCTCTCACACACACACA  AATGAAACCGTGTGCTGACC  ATAGGCTCGCAGGGATGATT  ATGGATGGACAGCAGAGAGC  AGCCACTTGAGCGAGGACT |

F: forward primer, R: reverse primer.
