# Supplementary material for: Mediator MED23 regulates inflammatory responses and liver fibrosis
Source: PLoS Biol. 2019 Dec 5;17(12):e3000563. doi: 10.1371/journal.pbio.3000563 (PMC6917294; doi:10.1371/journal.pbio.3000563)
Supplement: S4 Table — qRT-PCR, quantitative real-time PCR. (DOCX) [file pbio.3000563.s011.docx]

**S4 Table. Primer sequences used in the qRT-PCR analysis**

| Primer name | Sequence (5’-3’) |
| --- | --- |
| *Med23*-F | TCGGAAAATCATTGGAGGAG |
| *Med23*-R | CAATAGGCAGGCATTTCGTT |
| *Acta2-*F | ACTGGGACGACATGGAAAAG |
| *Acta2-*R | GTTCAGTGGTGCCTCTGTCA |
| *Desmin-*F | AGCTCAAGTCA TCGCCCTTC |
| *Desmin-*R | GCAGA TCCCAACACCCTCTC |
| *Col1a1*-F | GCTCCTCTTAGGGGCCACT |
| *Col1a1*-R | CCACGTCTCACCATTGGGG |
| *Col3a1*-F | GGGGACCAGGGCGACCACT |
| *Col3a1*-R | CAGGTGAACCCGGCAAGAACG |
| *Mmp9-*F | ACCACAGCCAACTATGACCAGGAT |
| *Mmp9-*R | AAGAGTACTGCTTGCCCAGGAAGA |
| *Mmp13-*F | CTTTGGCTTAGAGGTGACTGG |
| *Mmp13-*R | AGGCACTCCACA TCTTGGTTT |
| *Timp-1-*F | GCATCTCTGGCATCTGGCATC |
| *Timp-1-*R | GGTATAAGGTGGTCTCGTTGA |
| *Timp-2-*F | GGGACACGCTTAGCATCACCC |
| *Timp-2-*R | GAAGAACTTGGCCTGGTGCCC |
| *Tgfβ1-*F | TGCGCTTGCAGAGATTAAAA |
| *Tgfβ1-*R | CTGCCGTACAACTCCAGTGA |
| *Tgfβr1-*F | CCGCCTTTAAGTAGTTCTGTTCGT |
| *Tgfβr1-*R | AGCCGTGGGGTCCTTTCTGTGC |
| *Pdgfβ-*F | CCCACAGTGGCTTTTCATTT |
| *Pdgfβ-*R | GTGGAGGAGCAGACTGAAGG |
| *Pdgfrβ-*F | TGGCCTCTGAGGACTAAAGC |
| *Pdgfrβ-*R | AACAGAAGACAGCGAGGTGG |
| *cIAP1-*F | CGATGCAGAAGACGAGATGA |
| *cIAP1-*R | TTTGTTCTTCCGGATTAGTGC |
| *cIAP2-*F | GAAGAAAATGCTGACCCTACAGA |
| *cIAP2-*R | GCTCATCATGACGACATCTTC |
| *Hgf-*F | TTCCCAGCTGGTCTATGGTC |
| *Hgf-*R | TGGTGCTGACTGCATTTCTC |
| *Cyclin D1-*F | GCGTACCCTGACACCAATCTC |
| *Cyclin D1-*R | CTCCTCTTCGCACTTCTGCTC |
| *c-Myc-*F | GTCTTTCCCTACCCGCTCA |
| *c-Myc-*R | CCTCATCTTCTTGCTCTTCTTCA |
| *c-Fos-*F | CCAGTCAAGAGCATCAGCAA |
| *c-Fos-*R | AAGTAGTGCAGCCCGGAGTA |
| *c-Jun-*F | TCCCCTATCGACATGGAGTC |
| *c-Jun-*R | TGAGTTGGCACCCACTGTTA |
| *Cd45-*F | CCAGCAGACAGGGTTGTTCT |
| *Cd45-*R | CGGGATAGATGCTGGCGATG |
| *Cd3g-*F | TGGAGTTCGCCAGTCAAGAG |
| *Cd3g-*R | CATATTCCCGGTCCTTGAGGG |
| *F4/80-*F | CCATCCACTTCCAAGATGGGTTA |
| *F4/80-*R | TGCCATCAACTCATGATACCCT |
| *Tnfα-*F | CGTCAGCCGATTTGCTATCT |
| *Tnfα-*R | CGGACTCCGCAAAGTCTAAG |
| *Il6-*F | CTGCAAGAGACTTCCATCCAG |
| *Il6-*R | AGTGGTATAGACAGGTCTGTTGG |
| *Il1α-*F | ACGTCAAGCAACGGGAAGAT |
| *Il1α-*R | AAGGTGCTGATCTGGGTTGG |
| *Il1β-*F | GCCCATCCTCTGTGACTCAT |
| *Il1β-*R | AGGCCACAGGTATTTTGTCG |
| *Ccl2/Mcp1-*F | TTAAAAACCTGGATCGGAACCAA |
| *Ccl2/Mcp1-*R | GCATTAGCTTCAGATTTACGGGT |
| *Ccl4-*F | TTCCTGCTGTTTCTCTTACACCT |
| *Ccl4-*R | CTGTCTGCCTCTTTTGGTCAG |
| *Ccl5-*F | GCTGCTTTGCCTACCTCTCC |
| *Ccl5-*R | TCGAGTGACAAACACGACTGC |
| *Ccl7-*F | GCTGCTTTCAGCATCCAAGTG |
| *Ccl7-*R | CCAGGGACACCGACTACTG |
| *Cxcl10-*F | CCAAGTGCTGCCGTCATTTTC |
| *Cxcl10-*R | GGCTCGCAGGGATGATTTCAA |
| *Ccr1-*F | CTCATGCAGCATAGGAGGCTT |
| *Ccr1-*R | ACATGGCATCACCAAAAATCCA |
| *Ccr2-*F | TGGCTGTGTTTGCCTCTCTA |
| *Ccr2-*R | CCCTGTGCCTCTTCTTCTCA |
| *Ccr5-*F | TTTTCAAGGGTCAGTTCCGAC |
| *Ccr5-*R | GGAAGACCATCATGTTACCCAC |
| *Cxcr2-*F | ATGCCCTCTATTCTGCCAGAT |
| *Cxcr2-*R | GTGCTCCGGTTGTATAAGATGAC |
| *Rorα*-F | CAATCCAACCCAATCAAAGC |
| *Rorα*-R | CAACTCCCGCCATACACAC |
| *G9a-*F | GGCTGATGTGAGAGAGGATGA |
| *G9a-*R | GGAACTGAAGAAGGCGATGC |
| *Actin*-F | CTGGCTGGCCGGGACCTGACA |
| *Actin*-R | ACCGCTCGTTGCCAATAGTGATGA |
| *Gapdh*-F | AGGTCGGTGTGAACGGATTTG |
| *Gapdh*-R | TGTAGACCATGTAGTTGAGGTCA |

F: forward primer, R: reverse primer.
