# Supplementary material for: Mediator MED23 regulates inflammatory responses and liver fibrosis
Source: PLoS Biol. 2019 Dec 5;17(12):e3000563. doi: 10.1371/journal.pbio.3000563 (PMC6917294; doi:10.1371/journal.pbio.3000563)
Supplement: S1 Text — (PDF) [file pbio.3000563.s012.pdf]

S1 Text. Supporting methods.

Western blotting

Western blotting was performed as previously described [1]. The following primary antibodies were used for western blotting: anti-MED23 (Abcam, ab200351), anti-α-SMA (Abcam, ab124964), anti-GAPDH (Proteintech, 60004-1-Ig), anti-cleaved caspase 3 (Cell Signaling Technology, 9661), anti-γ-H2AX (Cell Signaling Technology, 9718), anti-CCL2 (Proteintech, 66272-1-Ig), and anti-CXCL10 (PeproTech, 500-P129).

Quantitative Real-time PCR (qRT-PCR)

Total RNA was extracted from cells or liver tissues using TRIzol (Thermo, 15596018). cDNA synthesis was carried out with a PrimeScript™ RT reagent kit with gDNA Eraser (Takara, RR047A), and qRT-PCR was then performed with technical triplicates using TB Green™ Premix Ex Taq™ (Tli RNaseH Plus) (Takara, RR420A) in an ABI QuantStudio 6 Real-time PCR machine. All expression values were normalized to those of *Gapdh*. The primers are listed in Supplementary Tables S1 in the Supplementary.

Serum ALT, AST, and albumin measurement

Blood was collected from mice after anesthesia with sodium pentobarbital and was allowed to clot for 2 hours at 4 °C. Then, the collected blood was centrifuged to obtain serum. AST, ALT, and albumin levels were measured with commercial kits (Shensuoyoufu, Shanghai, China) according to the manufacturer’s instructions.

MCD diet-induced liver fibrosis model

Eight-week-old male mice were fed with methionine- and choline-deficient (MCD) diet (TP 3005G, Trophic Animal Feed High-tech Co., Ltd., China) for 2 months to induce liver fibrosis as previously described [2]. The food in cage was refreshed every 3 days. All mice were euthanized and livers were collected for histological, biochemical, and molecular analyses.

1. Chu Y, Gomez Rosso L, Huang P, Wang Z, Xu Y, Yao X, et al. Liver Med23 ablation improves glucose and lipid metabolism through modulating FOXO1 activity. Cell research. 2014;24(10):1250-65. Epub 2014/09/17. doi: 10.1038/cr.2014.120. PubMed PMID: 25223702; PubMed Central PMCID: PMC4185346.
2. Wang X, Zheng Z, Caviglia JM, Corey KE, Herfel TM, Cai B, et al. Hepatocyte TAZ/WWTR1 Promotes Inflammation and Fibrosis in Nonalcoholic Steatohepatitis. Cell metabolism. 2016;24(6):848-62. doi: 10.1016/j.cmet.2016.09.016. PubMed PMID: 28068223; PubMed Central PMCID: PMCPMC5226184.
