# Supplementary material for: Mediator MED23 regulates inflammatory responses and liver fibrosis
Source: PLoS Biol. 2019 Dec 5;17(12):e3000563. doi: 10.1371/journal.pbio.3000563 (PMC6917294; doi:10.1371/journal.pbio.3000563)
Supplement: Attachment — Submitted filename: rebuttal letter.docx [file pbio.3000563.s014.docx]

*Gang Wang, Professor*

*School of Life Sciences*

*Fudan University*

*Shanghai 200438, China*

Sep. 17th, 2019

Dear Dr. Jiang,

Here we are submitting a revised version of our manuscript entitled "Mediator MED23 regulates inflammatory responses and liver fibrosis" (PBIOLOGY-D-19-01595R1).

We thank the Academic Editor and three reviewers for their constructive suggestions, and we have made all efforts to respond carefully to each of the comments by the editor and 3 reviewers. With the additional data we have generated, combined with the data in the original version, we feel that the paper is significantly improved. Here is a summary of the experiments we have performed, and our point-to-point response to the individual criticisms of each reviewer is attached to this letter.

We have completed the following experiments that the reviewers asked us to do:

a) analyzed a metabolic stress-induced liver fibrosis mouse model (in addition to the CCl_4_ model);
b) examined the expression pattern of *MED23* in normal, cirrhosis, and hepatocellular carcinoma (HCC) samples from the clinical database;
c) checked the expression levels of *Ccl5* and *Cxcl10* in isolated primary hepatocytes;
d) examined the serum ALT & AST levels and hepatic *Cyp* expression in acute CCl_4_ model (24h);
e) analyzed the necrosis in *Med23*-KO livers in acute CCl_4_ model (24h);
f) compared the *Hgf* expression in acute CCl_4_ model (24h) between WT and *Med23*-KO livers;
g) performed the western blot showing the efficiency of *RORα* knockdown;
h) analyzed MED23 binding at the *Ccl5* and *Cxcl10* locus and at genome-wide scale.

In summary, our paper describes for the first time that the Mediator MED23 plays an important role in liver fibrosis and provides new insight into the molecular mechanisms of inflammatory responses that initiate fibrotic changes upon liver injury. In the future, targeting the MED23/chemokine axis may provide intervention strategies for liver fibrosis. We hope that the changes in the revised manuscript meet with your approval. Your favorite consideration is truly appreciated.

Sincerely yours,

*Gang Wang, Professor*

*School of Life Sciences*

*Fudan University*

***Reviewer #1: Wang et al, investigated the role of the MED23 subunit of the transcriptional Mediator complex in CCl4-induced liver injury. Mice with hepatic Med23 deletion exhibited aggravated CCl4-induced liver fibrosis, enhanced inflammation and increased hepatocyte regeneration. The authors suggest that this phenotype is mediated by MED23-induced suppresses of orphan nuclear receptor RORα, which regulates the activation of liver fibrosis-related chemokines CCL5 and CXCL10.***

**1. *Half of the paper is focusing on the CCl4 responses in MED23-deleted mice that show increased HSC activation, fibrosis, inflammation and hepatocyte proliferation while hepatocyte apoptosis is reduced (Fig.1-4, half of the figures). While this data is relevant, most of this data is descriptive and may be related to the same mechanisms, meaning that it is likely looking at the same observation from different angles.***

Thanks for the reviewer’s comment. However, we respectfully disagree with the reviewer that “data in Figure 1-4 are mainly descriptive”, as we first observed the phenotype in Figure 1, then the cellular and molecular studies were carried out step-by-step and eventually led to the molecular and cellular understanding of the phenotype at last (from Figure 2 to 7).

Specifically, in Figure 1, through the chronic CCl_4_ treatment, we first observed that hepatic *Med23* deletion could aggravate the liver fibrosis characterized by increased HSCs activation and collage accumulation. In order to understand this phenotype, we then evaluated the liver cell death and hepatocyte proliferation (Figure 2), and found out that these cellular changes appeared not to account for the aggravated liver fibrosis. We then went on further dissecting the inflammation infiltration and showed that immune cells and a variety of cytokines and chemokines were significantly increased in *Med23*-KO livers compared to controls (Figure 3). These data prompted us to examine the effects of MED23 on the inflammatory response under the acute CCl_4_ treatment (24 hour). Interestingly, the acute CCl_4_ treatment resulted in increasing cytokines and chemokines in *Med23*-KO livers compared to controls (Figure 4). Following these experiments, we finally nailed down to two important fibrosis related chemokines, *Ccl5* and *Cxcl10,* which were up-regulated by hepatic *Med23*-deletion and may help to trigger the increased inflammation infiltration and liver fibrosis in *Med23*-deleted mice (Figure 5). We hope the reviewer can now be convinced that these are logic, step-by-step experiments leading to our discovery.

**2. *The paper relies on a single model and the rationale for studying MED23 is not well explained. The authors should employ more liver injury models to exclude that their findings are specific to CCl4, a standardized model for liver fibrosis research but without much human relevance. Moreover, they should analyze MED23 in patients. It is not clear at all whether MED23 activity is altered in human fibrosis and contributes to disease progression.***

**Rationale:** Our previous study revealed the important role of Mediator MED23 in obesity and diabetes (Chu et al., 2014), and we also have an ongoing project investigating the role of MED23 in liver cancer. Since liver fibrosis could accompany metabolic diseases, and may lead to liver cancer, so we decided to first examine the possible function of MED23 in liver fibrosis. In the revised manuscript, we have expanded the introduction to provide better explanation why we are particularly interested in studying Mediator MED23 subunit.

**Additional Model:** While preparing the first draft of our manuscript, we have already managed to establish another widely nonalcoholic steatohepatitis (NASH) model, which used methionine/choline-deficient (MCD) diet to induce liver pathology including steatosis, liver inflammation and **fibrosis**, and hepatocyte death (Wang et al., 2016). We have observed similar levels of liver weight, liver/body ratio, and plasma ALT & AST in the control and *Med23*-deleted mice after feeding MCD-diet for 2 months, except that the body weight was slightly increased in *Med23*-deleted mice (see Figure below, panel A, B). Similar to the CCl_4_-induced fibrosis model, *Med23* silencing also resulted in increasing mRNAs related to liver fibrosis, including *Col1a1*, *Col3a1*, *Tgfβ1*, *Pdgfβ*, *Tgfβr1*, *Pdgfrβ*, and *Timp1*. However, the hepatic inflammation markers were largely unchanged, except the increased *Tnfα* in *Med23*-KO livers (see Figure below, panel C, D). Importantly we observed that there was an increase in α-SMA^+^ cells (see Figure below, panel E, F) as well as protein level of α-SMA and Col1a1 in *Med23*-deficient mouse livers (see Figure below, panel G, H). Taken together, these results suggest that *Med23* ablation can promote MCD-diet induced liver fibrosis, although the underlying molecular mechanisms in this model may be different from the CCl_4_-induced fibrosis model.

(A) Analysis of liver weight, body weight, and liver/body weight in *med23*^f/f^ and *med23*^Δli^ mice fed with MCD-diet. (B) Serum ALT and AST were measured in *med23*^f/f^ and *med23*^Δli^ mice fed with MCD-diet. (C-D) qRT-PCR analysis of fibrosis-related factors (C) and inflammation-related factors (D) in whole liver extracts of *med23*^f/f^ and *med23*^Δli^ mice fed with MCD-diet. (E) Liver sections from *med23*^f/f^ and *med23*^Δli^ mice fed with MCD-diet were stained with H&E and α-SMA, and representative views were shown. (F) Quantification of the α-SMA-positive area in livers of *med23*^f/f^ and *med23*^Δli^ mice. (G) The total protein was extracted from whole livers of *med23*^f/f^ and *med23*^Δli^ mice fed with MCD-diet and analyzed by western blotting using the indicated antibodies. HSP70 was used as a loading control. (H) Quantification of the α-SMA and Col1a1 levels in livers of *med23*^f/f^ and *med23*^Δli^ mice (by the gray degree value in G) and normalized to the HSP70. Error bars denote SEM from three independent experiments. n = 6 for each genotype. Data are presented as mean ± SEM. **P* < 0.05, ***P* < 0.01, ****P* < 0.001

**Clinical Data:** To examine whether MED23 is involved in human patients with liver fibrosis, we analyzed the expression pattern of MED23 in normal, cirrhosis, and hepatocellular carcinoma (HCC) samples from the published clinical database (GSE14323). The result showed comparable *MED23* mRNA levels between cirrhosis and normal samples (see Figure below), suggesting that the liver fibrosis was not simply regulated by differential expression levels of MED23. This is consistent with the notion that Mediator complex mainly acts as the co-factor by modulating the activity of TFs to regulate the biological process (Yin and Wang, 2014), and the co-factor mRNA levels are not necessarily altered in the disease state. MED23 could participate in the liver diseases in many other ways, including modulating the activities of the involved transcription factors, which is exactly what we have found in this study.

The published database (GSE14323) (Mas et al., 2009) was utilized to evaluate the *MED23* expression.

**3. *The authors do not sufficiently investigate whether MED23 may simply regulate responses to CCl4 and liver injury. The authors show ALT levels in the chronic CCl4 model, where liver injury is typically low towards the end of experiments and where differences may be difficult to reveal. There is no data on serum ALT in the acute injury models and not investigation of key factors that regulate CCl4 metabolization and liver injury such as Cyp expression/activity. Moreover, the assessment of apoptosis by TUNEL assay and cleaved caspase is not highly relevant as most of CCl4-induced cell death is necrosis. In light of these, it would also have been advantageous to use additional models that do not rely on biactivation of a toxin and/or mostly necrotic cell death (see comments above).***

Per the reviewer’s suggestion, we have measured the serum levels of ALT and AST in acute CCl_4_ model, and found that serum ALT and AST levels were both dramatically reduced in *Med23*-KO mice compared to that in *med23*^f/f^ mice after acute CCl_4_ treatment (now included in the revised Fig S2B and Figure below, panel A), suggesting the decreased liver injury after *Med23* deletion. This is consistent with the observed phenotype of less cell death and less DNA damage (indicated by γ-H2AX) in *Med23*-KO than in WT livers (Figure 4C).

We also analyzed the *Cyps* expression profile through RNA-seq of the acute CCl_4_-treated livers and found that many *Cyps* were significantly increased after hepatic *Med23* deletion, while some *Cyps* were reduced (see Figure below, panel B). We thought that the differential *Cyps* gene expression certainly correlated with the *Med23*-deficiency and liver injury, however, the causal relationship between the altered *Cyps* profile and the liver fibrosis is too complicated. Future studies are needed in understanding how Mediator regulates the *Cyps* expression and drug metabolism.

We agree with the reviewer that necrosis but not apoptosis is more relevant to the CCl_4_-induced liver injury, and it is known that CCl_4_-induced hepatotoxicity is characterized by centrilobular necrosis (Wong et al., 1998). We examined our histological staining data, and observed that the centrilobular necrosis only occurred in the WT but not in the *Med23*-KO livers after acute CCl_4_ treatment (now included in the revised Fig S2A and Figure below, panel C, also Figure 4A). Necroptosis is a programmed form of necrosis marked by phosphorylation of MLKL (Sun et al., 2012). We checked the phosphorylated MLKL in CCl_4_-treated livers and found no phosphorylated MLKL, indicating the absence of necroptosis in both the WT and *Med23*-KO livers (see Figure below, panel D). Therefore, the necrosis seems not to contribute to the aggravated CCl_4_-induced fibrosis in *Med23*-KO mice.

Regarding the additional model that does not rely on bioactivation of a toxin and/or mostly necrotic cell death, we did investigate the metabolic stress-induced liver fibrosis model with MCD diet. For details, please see our response to the previous question.

(A) Serum ALT and AST were measured in *med23*^f/f^ and *med23*^Δli^ mice after acute CCl_4_ treatment for 24 h. (B) Heatmap analysis of differential expression genes of *Cyps* between *med23*^f/f^ and *med23*^Δli^ mouse livers after acute administration of CCl_4_ for 24 hours. We found that 20 genes were upregulated (>2-fold) and 11 genes were downregulated (>2-fold) in the *med23*^Δli^ mice. (C) Liver sections from *med23*^f/f^ and *med23*^Δli^ mice were stained with H&E and representative views were shown. (D) The total protein was extracted from whole livers of *med23*^f/f^ and *med23*^Δli^ mice and analyzed by western blotting using the indicated antibodies. L-029 cells treated with TNFα and Z-VAD-FMK was used as a positive control of programmed necrosis. HSP70 was used as a loading control. n = 4-5 for each genotype. Data are presented as mean ± SEM. **P* < 0.05

**4. *The clodronate and in vitro experiments do not conclusively prove a role for MED23 via the regulation of hepatocyte Ccl5 and Cxcl10. The authors should have analyzed this in primary hepatocytes and possbily used single cell RNA-seq to compare Ccl5 and Cxcl10 expression between MED23 and floxed controls as well as between hepatocytes and other cell types in the MED23-deleted mice - this way they could have strengthened their hypothesis that hepatocytes represent the main Ccl5- and Cxcl10-secreting cell type in mice with MED23 in hepatocytes. Finally, it would have been important to show the role of Ccl5 and/or Cxcl10 by deleting these in hepatocytes in vivo - which can nowadays be achieved by hepatocyte-specific silencing or deletion methods without lengthy breedings.***

We have isolated primary hepatocytes from control and *Med23*-KO mice with acute CCl_4_ treatment for 24 h, and the cells were collected for RNA extraction once they adhered to the dishes. The mRNA level of *Ccl5* and *Cxcl10* seems to be consistently higher in primary hepatocytes with *Med23-*deletion compared to that in the controls (see Figure below), which is consist with the results of *in vivo* clodronate treatment and *in vitro* experiments using AML12 cells. This result again supported the notion that *Med23* deficiency enhanced the *Ccl5* and *Cxcl10* expression in hepatocytes. For the limited time and budget, we did not go further to perform the single cell analysis.

qRT-PCR analysis of genes in primary hepatocytes of *med23*^Δli^ and control mice acutely

treated with CCl_4_ for 24 h. n = 3 for each genotype. Data are presented as mean ± SEM.

It happens that the critical roles of *Ccl5* and/or *Cxcl10* in liver fibrosis have been comprehensively investigated (Berres et al., 2010; Hintermann et al., 2010; Seki et al., 2009; Tomita et al., 2016), and we have now emphasized the related literatures in the revised introduction. Briefly, deletion of CCL5 receptor (CCR5) can prevent liver fibrosis (Seki et al., 2009). In the same study, remarkable upregulation of CCL5 and CCR5 were detected in patients with hepatic cirrhosis. In addition, Berres et al. showed that CCL5-knockout mice displayed reduced hepatic fibrosis following CCl_4_ injection or feeding on a methionine and choline-deficient (MCD) diet (Berres et al., 2010). Moreover, two studies demonstrated that CXCL10 seems to be profibrogenic, either by modulating hepatic macrophage infiltration or by inhibiting NK cell-mediated HSC inactivation (Hintermann et al., 2010; Tomita et al., 2016). Collectively, these literatures have demonstrated that *Ccl5* and/or *Cxcl10* are essential for the pathogenesis of liver fibrosis in different models; and both genetic deletion and pharmacological inhibitors to these targets could attenuate the development of liver fibrosis. Considering these literatures knowledge, we did not go further to manipulate these two chemokines in our mouse model.

**5. *The authors found that Hgf, was higher in MED23-deleted livers than in control livers. It is not clear why this is the case as HGF is normally expressed by stellate cells and endothelial cells. As Hgf is an important modulator of liver injury and regeneration, this could also represent a major mechanism by which MED23 regulates responses to liver injury, rather than the postulated Ccl5 and Cxcl10 pathway in hepatocytes.***

In the manuscript, we found that *Hgf* was higher in *Med23*-KO livers than in control livers after long-term CCl_4_ treatment for 1 month (Figure 2E). Since HGF is secreted by non-parenchymal liver cells (Matsumoto and Nakamura, 1991), we thought that the increased *Hgf* in *Med23*-KO livers is likely from the increased non-parenchymal liver cells, including the activated hepatic stellate cells and Kupffer cells (Figure 1 and Figure 3). By contrast, in the acutely exposure model (CCl_4_, 24 h), we failed to observe the increased hepatic *Hgf* expression in *Med23*-KO mice compared to that in control mice (see Figure below). Taken together, the *Hgf* up-regulation at one month (Figure 2E) is unlikely caused by hepatic *Med23* deletion directly, however, it is possible that the higher HGF may act in a paracrine manner to stimulate hepatocyte division and liver regeneration (Figure 2).

In general, higher HGF inhibits liver fibrosis (Cao et al., 2017; Ueki et al., 1999; Xia et al., 2006); therefore HGF doesn’t act like the downstream effector of *Med23*-deficiency that leads to liver fibrosis. Instead, through *in vivo* and *in vitro* experiments (Figure 5), we identified two important fibrosis related chemokines, *Ccl5* and *Cxcl10,* which were regulated by MED23 and RORα, and can trigger the increased inflammation infiltration and liver fibrosis in *Med23*-KO mice.

qRT-PCR analysis of *Hgf* expression in whole liver extracts of control

and *med23*^Δli^ mice (n = 5). Data are presented as mean ± SEM.

**Minor points**

**1. *The authors state “the expression of Desmin, another marker of HSCs activation, was also higher in med23^Δli^ mouse livers than in med23^f/f^ mouse livers”. Desmin is not a marker of activation - it is a marker of HSC and an increase may reflect an increased number of HSC in injured livers.***

Thanks for the reviewer’s suggestion, the statement was modified to “the expression of *Desmin*, a marker of HSCs reflecting the number of HSCs, was also higher in *med23*^Δli^ mouse livers than in *med23*^f/f^ mouse livers” and added into the revised manuscript.

***Reviewer #2: Liver fibrosis is a wound-healing response that is a consequence of chronic liver diseases. Despite the widespread of this disease, the underlying molecular mechanisms remain largely unknown. Development of novel effective therapies for liver fibrosis requires identifying effective molecular targets. The focus of this research is the study of the molecular mechanism underlying liver fibrosis that is a relevant and important issue.***

***The manuscript “The Mediator subunit MED23 regulates inflammatory responses and liver fibrosis” is dedicated to studying of the role MED23 subunit of the Mediator complex in the development of experimental liver fibrosis. The authors showed that mice with hepatic Med23 deletion exhibited aggravated CCl4-induced liver fibrosis. The manuscript provides evidence that MED23 negatively regulates the expression of the hepatic chemokines CCL5 and CXCL10 via suppressing the activity of the retinoid-related orphan nuclear receptor, RORα.***

***In summary, this manuscript is of value for the further development of our knowledge of the molecular mechanism underlying liver fibrosis and will help the development of more effective treatment options. I would recommend the manuscript for publication, but I have the following comments.***

***CRITIQUE***

***My main problem with this manuscript is that the authors made several deficiencies, which cast doubt on the conclusions and correctness of the model proposed.***

**1. *Necessary to give data about the number of mice in all experiments.***

We appreciate the reviewer’s suggestion. We have now included the number of mice in the figure legends of all experiments.

**2. *Fig 6F. Necessary to show Western-Blot for proving the effectivity of the knocked down Rora in protein level.***

Unfortunately, the good antibody to RORα from Santa Cruz (as seen in the literature) is out of stock now. Our antibody against RORα (from Proteintech) is not in good quality. We did try our best in performing the western blot for proving the effectivity of the RORα knockdown (see Figure below), and the knockdown effect seems to be just OK.

**3. *Fig 6I. Necessary to describe this experiment with all details in Materials and Methods. I have doubts about the conclusions made by the authors. Controls have significant differences. It should be discussed in the manuscript with an explanation. In the current moment, conclusion about a functional interaction between RORα and MED23 in controlling Ccl5 and Cxcl10 expression needs additional experiments.***

We have now carefully described the experiment of Figure 6I in Materials and Methods of revised manuscript as the following: For “**siRNA transfection:** AML12 cells were transfected with the indicated siRNAs (10 μM) using RNAiMax transfection reagent (Invitrogen, 13778150) according to the manufacturer’s instructions. Forty-eight hours after transfection, cells were collected for protein and RNA extraction. For Figure 6I, retrovirally-mediated *Med23* knockdown or the control AML12 cells were seeded into 6-well plate (150000 cells /well). Next day these cells were transfected with the control siRNA or RORα siRNA (10 μM) using RNAiMax transfection reagent. Forty-eight hours after transfection, cells were collected for protein and RNA extraction.” We have also relabeled the Figure 6I, trying to make it clearer.

Regarding “***Controls have significant differences”*** and the possible “***functional interaction between RORα and MED23 in controlling Ccl5 and Cxcl10 expression”,*** we thought we have the following explanation: (i) in the Figure 6I controls (dark bars), in which the cells were virally-infected without sh*Med23*, si-*RORα* knockdown greatly reduces the expression of *Ccl5* and *Cxcl10*. This is consistent with the data in Figure 6E; (ii) also in Figure 6I, in the shMed23 AML12 cells, *Ccl5* and *Cxcl10* were increased as expected (also shown in Figure 5E), which was almost abolished following transfection with RORα siRNA (10 μM); (iii) the residual increase of *Ccl5* and *Cxcl10* upon RORα and MED23 double-knockdown might be due to the incomplete deficiency of RORα mediated by siRNA; (iv) lastly, we have tried but failed to detect the direct interaction between MED23 and RORα by co-IP, which might be due to the transient interaction nature or additional factor(s) involved.

Since other additional assays also support that MED23 could modulate the transcriptional activity of RORα negatively (Figure 6J, & 6K), we attempted to conclude that RORα and MED23 act together in controlling *Ccl5* and *Cxcl10* expression in the development of liver fibrosis. Considering the reviewer’s concern, we have toned down our conclusion by stating that MED23 and RORα may possibly have a functional interaction.

**4. *Fig 6J. The dual luciferase reporter assay should be described in Materials and Methods.***

The dual luciferase reporter assay was carefully described in Materials and Methods now.

**5. *Fig 6K. This experiment should be described with details in the manuscript.***

Fig 6K experiment have been described in details in the revised manuscript and as following:

“Moreover, we transfected HeLa cells with increasing dosage of *RORα* and *Med23* along with RORE reporter to further verify the regulation of RORα activity by MED23. Increasing RORα expression up-regulates the RORE reporter activity in a dose-dependent manner (Figure 6K). Importantly, RORα-activated RORE activity was repressed gradually with increasing dosage of *Med23* (up to 2-fold decrease) (Figure 6K).”

**6. *Fig 7A. Necessary to show a base level of signal for ChIP on non-promoter site of the genome.***

We have now analyzed the binding of MED23 and Pol II at additional sites including a distal promoter region (P3) and two coding regions (P4 and P5) of *Ccl5* and *Cxcl10* genes (now included in the revised Fig 7A and Figure below, panel A). The binding pattern of MED23 and Pol II at the *Ccl5* and *Cxcl10* locus were similar, and importantly, both the occupancy of MED23 and Pol II peaked at the core promoter regions (P1 and P2) (now included in the revised Fig 7A and Figure below, panel A). Interestingly we also observed the occupancy of MED23 in the *Ccl5* coding region (less than that in the core promoter regions), implying that MED23 might also affect *Ccl5* expression via the post-recruitment steps such as mRNA processing as we previously reported (Huang et al., 2012; Wang et al., 2005; Wang et al., 2013). Also this is consistent with the previous literature that Mediator does bind to some gene coding regions (Zhu et al., 2006).

Since our lab has ongoing efforts performing ChIP-seq assay using MED23 antibody (from Abcam), we also analyzed the genome-wide binding profile of MED23 in the AML12 cells, and found that *Med23*-deletion greatly reduced the MED23 enrichment at TSS globally (see Figure below, panel B & C), indicating the good efficiency of this antibody for ChIP assay. By zooming in the ChIP-seq data, we observed the enrichment of MED23 at the promoter regions of *Ccl5* and *Cxcl10*, (see Figure below, panel D); and, again MED23 binds to *Ccl5 gene* coding region (see Figure below, panel D). Importantly, all these MED23-binding were reduced by *Med23*-knockdown, indicating that our data are in reasonably good quality.

(A) ChIP analysis of MED23 and Pol II occupancy in AML12 cells. The precipitated DNA was analyzed by qRT-PCR with primers targeting the indicated regions of *Ccl5* and *Cxcl10*. The relative binding level of each factor was calculated by normalization to the input DNA. (B) Genome-wide occupancy of MED23 in shCtrl (green) and shMed23 (orange and red) AML12 cells. (C) ChIP-seq density heatmaps of MED23 were shown within the extended gene regions (-3 kb of TSS to +3 kb of TTS) of total genome-wide genes. (D) Relative quantification of total RPM (reads per million) normalized to input on previously annotated region. Data are presented as mean ± SEM. **P* < 0.05, ***P* < 0.01

**7. *I would like to ask the authors to provide more discussion in the text. I highly recommend expanding the Introduction part and give more information about the factors used in the work.***

More discussions are included in the revised manuscript regarding the epigenetic mechanisms involved in transcription regulation by Mediator and RORα. Further, we have expanded the introduction part and given more information about the factors, especially Mediator complex and RORα.

**8. *In this manuscript, a significant part of the data is the results of the qRT-PCR. I would like to ask the authors to pay more intention for presenting this type of data. Nowadays good practice to use two or three genes for normalization especially if the differences between experiment and control less than 2-folds***.

We thank the reviewer for this suggestion, and we have verified our qRT-PCR data in two ways:

(1) We first analyzed the heatmap of the RNA-Seq data on CCl_4_-treated livers (24h), and revealed that the FPKM of multiple cytokines and chemokines were increased in *Med23*-KO compared to WT livers (see Figure below), which are in good agreement with our qRT-PCR data.

(2) We also re-analyzed the expression of critical genes in Figure 1-3 by normalizing the mRNA to *36B4*, a constitutively-expressed ribosomal gene (see Figure below), and found that the newly normalized results were similar with our data normalized to *Gapdh* in the manuscript. Therefore, we are confident that our qRT-PCR data are reliable and convincing.

(A-D) qRT-PCR analysis of genes expression in whole liver extracts of control and *med23*^Δli^ mice following CCl_4_ injection 1 month (n = 6 for each genotype). Data are presented as mean ± SEM.

**P* < 0.05, ***P* < 0.01, ****P* < 0.001

**Reviewer #3: *The authors study the role of Med23 in experimental models of liver fibrosis (carbon tetrachloride) in conditional knock out mice using Albumin-CRE, Med23 flox/flox mice. The authors find that deletion of Med23 in hepatocytes and cholangiocytes leads to increased liver fibrosis, decreased apoptosis but increased hepatocyte proliferative activity and increased inflammatory activity, though the characterization of inflammatory cell composition remains a bit superficial. In well designed in vitro studies using murine hepatocyte cell line AML12 and other models, the authors show that Med23 cooperates with RORα in negatively regulating cytokines CCL5 and CXCL10. The authors show that Med23 deletion causes decreased H3K9me2 occupancy among the promoter of CCL5 and CXLC10 thus identifying novel mechanism for Med23.***

***Though the authors ultimately do not show the biological relevance of Med23-CCL5/CXLC10 axis in liver fibrosis and inflammation e.g. by showing abrogation of increased liver fibrosis in double knock out mice of both Med23 and CCL5/CXCL10 or data on Med23 expression levels in human liver datasets from public databases (Suppl. Fig. 3), the shown data are of very good quality and experiments are well designed. The conclusions are sound and the manuscript well written with the topic within the journals scope, I thus recommend the acceptance of the manuscript for publication.***

We thank the reviewer for all the comments. Regarding the further genetic manipulation of CCL5/CXCL10 in our mouse model, we feel that the critical roles of CCL5 and CXCL10 in liver fibrosis have been investigated comprehensively (Berres et al., 2010; Hintermann et al., 2010; Kizu et al., 2015; Li et al., 2017; Seki et al., 2009; Tomita et al., 2016; Zhang et al., 2014). Related literatures have been described in the introduction in the revised manuscript. Briefly, Berres et al. showed that CCL5-knockout mice displayed reduced hepatic fibrosis following CCl_4_ injection (Berres et al., 2010); and two studies demonstrated that CXCL10 seems to promote liver fibrosis by modulating hepatic inflammatory responses (Hintermann et al., 2010; Tomita et al., 2016). Therefore, both CCL5 and CXCL10 have been shown to be important for the pathogenesis of liver fibrosis in animal models. Considering these literatures and for the limited time being, we did not go further to manipulate these two chemokines in our mouse model.

Per the reviewer’s suggestion, we analyzed the expression pattern of MED23 in normal, cirrhosis, and hepatocellular carcinoma (HCC) samples from the published clinical database (GSE14323). The result showed comparable *MED23* mRNA levels between cirrhosis and normal samples (see Figure below), suggesting that the liver fibrosis was not simply regulated by differential expression levels of MED23. This is consistent with the notion that Mediator complex mainly acts as the co-factor by modulating the activity of TFs to regulate the biological process (Yin and Wang, 2014), and the co-factor mRNA levels are not necessarily altered in the disease state. MED23 could participate in the liver diseases in many other ways, including modulating the activities of the involved transcription factors, which is exactly what we have found in this study.

The published database (GSE14323) (Mas et al., 2009) was utilized to evaluate *MED23* expression

***Minor comments:***

***additional information regarding the catalog numbers of siRNA / plasmids used to knock down targets would be informative for readers interested in replicating the studies.***

The sequences of shRNA oligonucleotides for virally-targeting Med23 have also been included in the supporting information (S1 Table). The siRNA oligonucleotides were synthesized by GenePharma (Shanghai), and the related sequence information has now been included in the supporting information (S2 Table).

**Response references:**

Berres, M.L., Koenen, R.R., Rueland, A., Zaldivar, M.M., Heinrichs, D., Sahin, H., Schmitz, P., Streetz, K.L., Berg, T., Gassler, N.*, et al.* (2010). Antagonism of the chemokine Ccl5 ameliorates experimental liver fibrosis in mice. The Journal of clinical investigation *120*, 4129-4140.

Cao, Z., Ye, T., Sun, Y., Ji, G., Shido, K., Chen, Y., Luo, L., Na, F., Li, X., and Huang, Z. (2017). Targeting the vascular and perivascular niches as a regenerative therapy for lung and liver fibrosis. Science translational medicine *9*, eaai8710.

Chu, Y., Gomez Rosso, L., Huang, P., Wang, Z., Xu, Y., Yao, X., Bao, M., Yan, J., Song, H., and Wang, G. (2014). Liver Med23 ablation improves glucose and lipid metabolism through modulating FOXO1 activity. Cell research *24*, 1250-1265.

Hintermann, E., Bayer, M., Pfeilschifter, J.M., Luster, A.D., and Christen, U. (2010). CXCL10 promotes liver fibrosis by prevention of NK cell mediated hepatic stellate cell inactivation. J Autoimmun *35*, 424-435.

Huang, Y., Li, W., Yao, X., Lin, Q.J., Yin, J.W., Liang, Y., Heiner, M., Tian, B., Hui, J., and Wang, G. (2012). Mediator complex regulates alternative mRNA processing via the MED23 subunit. Molecular cell *45*, 459-469.

Kizu, T., Yoshida, Y., Furuta, K., Ogura, S., Egawa, M., Chatani, N., Hamano, M., Takemura, T., Ezaki, H., Kamada, Y.*, et al.* (2015). Loss of Gab1 adaptor protein in hepatocytes aggravates experimental liver fibrosis in mice. Am J Physiol Gastrointest Liver Physiol *308*, G613-624.

Li, B.H., He, F.P., Yang, X., Chen, Y.W., and Fan, J.G. (2017). Steatosis induced CCL5 contributes to early-stage liver fibrosis in nonalcoholic fatty liver disease progress. Transl Res *180*, 103-117 e104.

Mas, V.R., Maluf, D.G., Archer, K.J., Yanek, K., Kong, X., Kulik, L., Freise, C.E., Olthoff, K.M., Ghobrial, R.M., McIver, P.*, et al.* (2009). Genes involved in viral carcinogenesis and tumor initiation in hepatitis C virus-induced hepatocellular carcinoma. Mol Med *15*, 85-94.

Matsumoto, K., and Nakamura, T. (1991). Hepatocyte growth factor: molecular structure and implications for a central role in liver regeneration. Journal of gastroenterology and hepatology *6*, 509-519.

Seki, E., De Minicis, S., Gwak, G.Y., Kluwe, J., Inokuchi, S., Bursill, C.A., Llovet, J.M., Brenner, D.A., and Schwabe, R.F. (2009). CCR1 and CCR5 promote hepatic fibrosis in mice. The Journal of clinical investigation *119*, 1858-1870.

Sun, L., Wang, H., Wang, Z., He, S., Chen, S., Liao, D., Wang, L., Yan, J., Liu, W., Lei, X.*, et al.* (2012). Mixed lineage kinase domain-like protein mediates necrosis signaling downstream of RIP3 kinase. Cell *148*, 213-227.

Tomita, K., Freeman, B.L., Bronk, S.F., LeBrasseur, N.K., White, T.A., Hirsova, P., and Ibrahim, S.H. (2016). CXCL10-Mediates Macrophage, but not Other Innate Immune Cells-Associated Inflammation in Murine Nonalcoholic Steatohepatitis. Scientific reports *6*, 28786.

Ueki, T., Kaneda, Y., H, Nakanishi, K., Sawa, Y., Morishita, R., Matsumoto, K., Nakamura, T., Takahashi, H., Okamoto, E., and Fujimoto, J. (1999). Hepatocyte growth factor gene therapy of liver cirrhosis in rats. Nature medicine *5*, 226-230.

Wang, G., Balamotis, M.A., Stevens, J.L., Yamaguchi, Y., Handa, H., and Berk, A.J. (2005). Mediator requirement for both recruitment and postrecruitment steps in transcription initiation. Molecular cell *17*, 683-694.

Wang, W., Yao, X., Huang, Y., Hu, X., Liu, R., Hou, D., Chen, R., and Wang, G. (2013). Mediator MED23 regulates basal transcription in vivo via an interaction with P-TEFb. Transcription *4*, 39-51.

Wang, X., Zheng, Z., Caviglia, J.M., Corey, K.E., Herfel, T.M., Cai, B., Masia, R., Chung, R.T., Lefkowitch, J.H., Schwabe, R.F.*, et al.* (2016). Hepatocyte TAZ/WWTR1 Promotes Inflammation and Fibrosis in Nonalcoholic Steatohepatitis. Cell metabolism *24*, 848-862.

Wong, F.W., Chan, W.Y., and Lee, S.S. (1998). Resistance to carbon tetrachloride-induced hepatotoxicity in mice which lack CYP2E1 expression. Toxicology and applied pharmacology *153*, 109-118.

Xia, J.L., Dai, C., Michalopoulos, G.K., and Liu, Y. (2006). Hepatocyte Growth Factor Attenuates Liver Fibrosis Induced by Bile Duct Ligation. American Journal of Pathology *168*, 1500-1512.

Yin, J.W., and Wang, G. (2014). The Mediator complex: a master coordinator of transcription and cell lineage development. Development *141*, 977-987.

Zhang, X., Shen, J., Man, K., Chu, E.S., Yau, T.O., Sung, J.C., Go, M.Y., Deng, J., Lu, L., Wong, V.W.*, et al.* (2014). CXCL10 plays a key role as an inflammatory mediator and a non-invasive biomarker of non-alcoholic steatohepatitis. Journal of hepatology *61*, 1365-1375.

Zhu, X., Wiren, M., Sinha, I., Rasmussen, N.N., Linder, T., Holmberg, S., Ekwall, K., and Gustafsson, C.M. (2006). Genome-wide occupancy profile of mediator and the Srb8-11 module reveals interactions with coding regions. Molecular cell *22*, 169-178.
