# Supplementary material for: Mediator MED23 regulates inflammatory responses and liver fibrosis
Source: PLoS Biol. 2019 Dec 5;17(12):e3000563. doi: 10.1371/journal.pbio.3000563 (PMC6917294; doi:10.1371/journal.pbio.3000563)
Supplement: Attachment — Submitted filename: The new response .docx [file pbio.3000563.s015.docx]

*Gang Wang, Professor*

*School of Life Sciences*

*Fudan University*

*Shanghai 200438, China*

Nov. 12th, 2019

Dear Dr. Jiang,

Here we are submitting the 2^nd^ revision of our manuscript entitled "Mediator MED23 regulates inflammatory responses and liver fibrosis".

Thanks again for your advice and for the reviewer’s comments. Here is a summary of what we have done to address the remaining concerns, and our point-to-point response is attached to this letter.

To address the reviewer’s remaining comment, we have done the following:

a) included a brief description about the MCD model in the revised manuscript; the related data has been included in the Supporting Information (the new S1 Fig);
b) analyzed the related chemokine expression in hepatocytes through data-mining a recent literature that described single-cell RNA sequencing of human liver tissues;
c) revised the discussion section regarding the possible alternative molecular mechanisms in connecting the hepatic *Med23*-deficiency to liver fibrosis;

We hope that the changes in the revised manuscript meet with your approval. Your favorite consideration is truly appreciated.

Sincerely yours,

Gang Wang, Ph.D

Professor

School of Life Sciences

Fudan University

Response to Reviewer #1:

***1. The second model (MCD model) was not included in the actual paper and mechanisms seem to be different from the CCl4 model and not be mediated by the proposed inflammatory mechanism. Moreover, the MCD model is nowadays viewed as inadequate model of NASH fibrosis and other dietary models are preferred (but it is adequate for purely mechanistic studies on fibrosis)***

Per the reviewer’s suggestion, we have now included the results of MCD model in the Supporting Information (S1 Fig), and included the method related to this model in the Supporting methods. A brief description about this model has also been added to the revised manuscript.

The pathogenesis of liver diseases (including liver fibrosis) is very complicated, and it has been quite a challenge using the animal models to fully mimic the features of human liver diseases. For the time being, we are not able to analyze any additional animal models of liver fibrosis. Although the MCD model couldn’t fully mimic human NASH, it does show the accumulated lipid and oxidative stress, which lead to necrosis, inflammation and subsequent fibrosis in liver. Overall the MCD model largely support our phenotypic findings in the CCl_4_ model, despite the underlying mechanisms could be different.

***2. The authors have not convinced this reviewer that the main role of MED23 is not related to protection from injury. This appears to be THE driver of the phenotype - everything else is likely secondary unless proven otherwise by functional experiments (see also point below).***

We respectfully disagree with the reviewer that*“the main role of MED23 in liver fibrosis is related to protection from injury, and thus everything else including enlarged inflammation and fibrosis is likely secondary”.* Assuming the effect of *Med23* deletion on liver fibrosis is due to the protection from injury, the fibrosis should be decreased, not increased in *Med23*-KO mice, because the severity of fibrosis positively correlates with the degree of liver injury. However, in our case, we did observe less liver injury and more liver fibrosis in *Med23*-KO mice. Therefore, the aggravated fibrosis in *Med23*-KO mice is unlikely due to the injury protection by *Med23*-deficiency. Instead we have carefully nailed down to the CCL5/CXCL10-mediated inflammatory responses, which eventually lead to the increased fibrogenesis in *Med23*-KO liver.

***3. This reviewer is not convinced that hepatocyte-secreted CCl5 and CXCL10 are the main drivers of fibrogenesis in this model. First of all, hepatocytes are not thought to be the main source of these chemokines in the liver. Second of all, hepatocyte isolations from CCl4-treated livers are inherently contaminated with inflammatory cells. The suggestion of using single cell RNA-seq as a platform that can avoid these problems was not taken up by the authors. In the absence of single cell RNA-seq analysis, the authors would need to provide evicence for this unlikely hypothesis, e.g. by hepatocyte-specific silencing of CCl5 and CXCL10 (which can nowadays be achieved without crossing mice, e.g. by AAV8- or GalNAc-mediated silencing).***

As the reviewer pointed out, the hepatocytes are thought not to be the main source of these chemokines (including CCL5 and CXCL10) in the liver **under the normal state**. However, we did notice a recent literature describing a single-cell RNA sequencing analysis of 10,000 cells from normal human liver tissue (Aizarani et al., 2019). We performed the data mining in this literature, and found that, while CCL5 is abundantly expressed in NK, NKT and T cells, it was also highly expressed in a subset of hepatocyte population (see figure at the end, in black circle), and modestly expressed in majority of the hepatocytes (see figure at the end). Two additional chemokines, CXCL10 and CCL2, seem to be equally expressed in almost all types of human liver cells at a modest level.

In our study, we found that Mediator MED23 seems to be able to suppress chemokine expression under normal state, whereas hepatic *Med23*-deletion is able to relieve the suppression and increase the chemokine/cytokine expressions. Therefore, at least some chemokines can be derived from hepatocytes, especially when hepatic homeostasis is disrupted by manipulation like deletion of *Med23* combining with CCl_4_ treatment.

We do agree with the reviewer that we could not rule out the possibility that hepatic *Med23*-deficiency could trigger the release of CCL5/CXCL10 from other cellular resources such as NK, NKT, and T cells. We have now added statement in the discussion section regarding the possible alternative molecular mechanisms in connecting the hepatic *Med23*-deficiency to liver fibrosis.

Lastly, we did not further perform the hepatic-specific silencing experiments on CCL5/CXCL10, because the critical roles of CCL5 and/or CXCL10 in liver fibrosis have been comprehensively investigated (Berres et al., 2010; Hintermann et al., 2010; Kizu et al., 2015; Seki et al., 2009; Tomita et al., 2016). Both genetic deletion and pharmacological inhibitors to these targets could attenuate the development of liver fibrosis. Considering these literature, we did not go further to manipulate these two chemokines in our mouse model.

**References**

Aizarani, N., Saviano, A., Sagar, Mailly, L., Durand, S., Herman, J.S., Pessaux, P., Baumert, T.F., and Grun, D. (2019). A human liver cell atlas reveals heterogeneity and epithelial progenitors. Nature *572*, 199-204.

Berres, M.L., Koenen, R.R., Rueland, A., Zaldivar, M.M., Heinrichs, D., Sahin, H., Schmitz, P., Streetz, K.L., Berg, T., Gassler, N.*, et al.* (2010). Antagonism of the chemokine Ccl5 ameliorates experimental liver fibrosis in mice. The Journal of clinical investigation *120*, 4129-4140.

Hintermann, E., Bayer, M., Pfeilschifter, J.M., Luster, A.D., and Christen, U. (2010). CXCL10 promotes liver fibrosis by prevention of NK cell mediated hepatic stellate cell inactivation. J Autoimmun *35*, 424-435.

Kizu, T., Yoshida, Y., Furuta, K., Ogura, S., Egawa, M., Chatani, N., Hamano, M., Takemura, T., Ezaki, H., Kamada, Y.*, et al.* (2015). Loss of Gab1 adaptor protein in hepatocytes aggravates experimental liver fibrosis in mice. Am J Physiol Gastrointest Liver Physiol *308*, G613-624.

Seki, E., De Minicis, S., Gwak, G.Y., Kluwe, J., Inokuchi, S., Bursill, C.A., Llovet, J.M., Brenner, D.A., and Schwabe, R.F. (2009). CCR1 and CCR5 promote hepatic fibrosis in mice. The Journal of clinical investigation *119*, 1858-1870.

Tomita, K., Freeman, B.L., Bronk, S.F., LeBrasseur, N.K., White, T.A., Hirsova, P., and Ibrahim, S.H. (2016). CXCL10-Mediates Macrophage, but not Other Innate Immune Cells-Associated Inflammation in Murine Nonalcoholic Steatohepatitis. Scientific reports *6*, 28786.
